# A deep learning approach for automated diagnosis of pulmonary embolism on computed tomographic pulmonary angiography

**DOI:** 10.1186/s12880-022-00916-0

**Published:** 2022-11-11

**Authors:** Pranav Ajmera, Amit Kharat, Jitesh Seth, Snehal Rathi, Richa Pant, Manish Gawali, Viraj Kulkarni, Ragamayi Maramraju, Isha Kedia, Rajesh Botchu, Sanjay Khaladkar

**Affiliations:** 1 Dr D.Y. Patil Medical College, Hospital and Research Center, Pune, India; 2 DeepTek Medical Imaging Pvt. Ltd., Pune, India; 3 Department of Radiology, Mahatma Gandhi Mission Medical College and Hospital, Navi Mumbai, India; 4 Department of Radiology, Royal Orthopedic Hospital, Birmingham, UK

**Keywords:** Artificial intelligence, Pulmonary embolism, Computed tomographic pulmonary angiography, U-Net architecture

## Abstract

**Background:**

Computed tomographic pulmonary angiography (CTPA) is the diagnostic standard for confirming pulmonary embolism (PE). Since PE is a life-threatening condition, early diagnosis and treatment are critical to avoid PE-associated morbidity and mortality. However, PE remains subject to misdiagnosis.

**Methods:**

We retrospectively identified 251 CTPAs performed at a tertiary care hospital between January 2018 to January 2021. The scans were classified as positive (n = 55) and negative (n = 196) for PE based on the annotations made by board-certified radiologists. A fully anonymized CT slice served as input for the detection of PE by the 2D segmentation model comprising U-Net architecture with Xception encoder. The diagnostic performance of the model was calculated at both the scan and the slice levels.

**Results:**

The model correctly identified 44 out of 55 scans as positive for PE and 146 out of 196 scans as negative for PE with a sensitivity of 0.80 [95% CI 0.68, 0.89], a specificity of 0.74 [95% CI 0.68, 0.80], and an accuracy of 0.76 [95% CI 0.70, 0.81]. On slice level, 4817 out of 5183 slices were marked as positive for the presence of emboli with a specificity of 0.89 [95% CI 0.88, 0.89], a sensitivity of 0.93 [95% CI 0.92, 0.94], and an accuracy of 0.89 [95% CI 0.887, 0.890]. The model also achieved an AUROC of 0.85 [0.78, 0.90] and 0.94 [0.936, 0.941] at scan level and slice level, respectively for the detection of PE.

**Conclusion:**

The development of an AI model and its use for the identification of pulmonary embolism will support healthcare workers by reducing the rate of missed findings and minimizing the time required to screen the scans.

## Introduction

Pulmonary embolism (PE) is an emergency condition associated with a high mortality and morbidity rate. The annual incidence of embolism is approximately 60–70 cases per 1,00,000 people, resulting in up to 1,00,000—3,00,000 deaths annually [1]. PE is the third most common cause of cardiovascular disease events, with only myocardial infarction and stroke having a higher prevalence [2]. Cancer-associated PE imposes an additional burden on patients and healthcare systems. Incidental diagnosis of cancer in patients hospitalized for acute PE was associated with 90% increase in overall mortality, longer period of stay at hospitals, higher cost of hospitalization, and higher risk of rehospitalization and home health services on discharge from hospital [3]. On the other hand, PE is also incidentally diagnosed in approximately 5% of patients with cancers on routine medical imaging. Cancer patients with incidental PE are at higher risk of recurrent venous thromboembolism despite anticoagulant treatment [4]. The most common cause of death from PE is a failure to diagnose [5]. The diagnostic approach to PE usually involves a series of investigations, including echocardiography and D-dimer, establishing the necessity for further confirmatory examinations like Computed Tomographic Pulmonary Angiography (CTPA) and ventilation/perfusion scans [6]. Because of its convenience of operation, CTPA enables a definitive diagnosis of PE and has practically eliminated the use of ventilation/perfusion scans [7]. According to multiple published studies, CTPA has a sensitivity and specificity of approximately 90% for detecting thrombus in the main pulmonary and segmental arteries [8, 9]. CTPA has no contraindications and a low (0.3–1.8%) associated morbidity [8]. Because of COVID-19 pneumonia, the frequency of cases diagnosed with PE on CTPA has increased. Planquette et al. reported a 5% prevalence of PE in the total COVID-19-affected population and a 20% prevalence in the clinically suspected population with acute PE [10].

A test lacking a high degree of sensitivity results in a delay in the timely initiation of anticoagulant therapy for embolus-positive patients, consequently leading to a higher mortality rate. Early anticoagulation therapy has been documented to reduce the mortality rate from 30% to 8% [11]. However, the increase in the utilization of medical imaging has made a timely and reliable diagnosis of CTPE extremely difficult for the healthcare system and radiology practitioners. The usage of CTPA in emergency departments has increased over 27-fold in the last two decades [12, 13]. The increased use of cross-sectional imaging has disproportionately increased the workload in emergency settings, and fatigue from long work hours and overnight shifts may contribute to an increase in diagnostic errors [14–16]. Although CTPA has become the gold standard in diagnosing PE, accurate prediction of PE using the modern multidetector row CTPAs requires time and expertise in reading cross-sectional CT images [17, 18]. These challenges can be addressed by employing automated detection algorithms for diagnosing PE in clinical settings. The advantage of AI over other conventional methods for the detection and diagnosis of pathologies has made it an attractive option for radiologists and healthcare providers [19]. Applications of AI in medical imaging help automate and standardize the protocols, which saves time and effort, improves diagnostic performance, and optimizes the workflow of radiologists [20, 21]. A well-trained neural network can help healthcare practitioners by highlighting the exams that are positive for PE in the worklist, thereby accelerating the diagnosis and communication workflow [22]. Previous computer-aided detection (CAD) algorithms for the automatic identification of PE on CTPAs had low sensitivities [23–26]. Recent studies have focused on the use of CTPA imaging for PE diagnosis using deep learning and convolutional neural networks.

The objective of our study is to externally validate the performance of a 2D segmentation model with U-Net architecture for automated detection of PE in CTPAs using clinically relevant data from the CTPAs performed at a tertiary care hospital. In this study, we validate an externally developed deep learning model “DxPE AI Screen” for early detection of emboli on CTPA to provide radiologists with a tested high-performance tool to help triage patients with PE. This tool has the potential to enable rapid and reliable radiology reporting while minimizing the substantial disparities between general radiologists and subspecialty-trained radiologists in interpreting scans. The U-Net architecture is generally used to address segmentation problems, but we effectively utilized it to solve a classification task by deriving probabilistic output from segmentation output. Our approach of converting slice-level segmentation predictions to scan-level classification predictions resulted in a high area under the curve and a short processing time.

## Materials and methods

### Patient selection (external validation dataset)

**Fig. 1:**
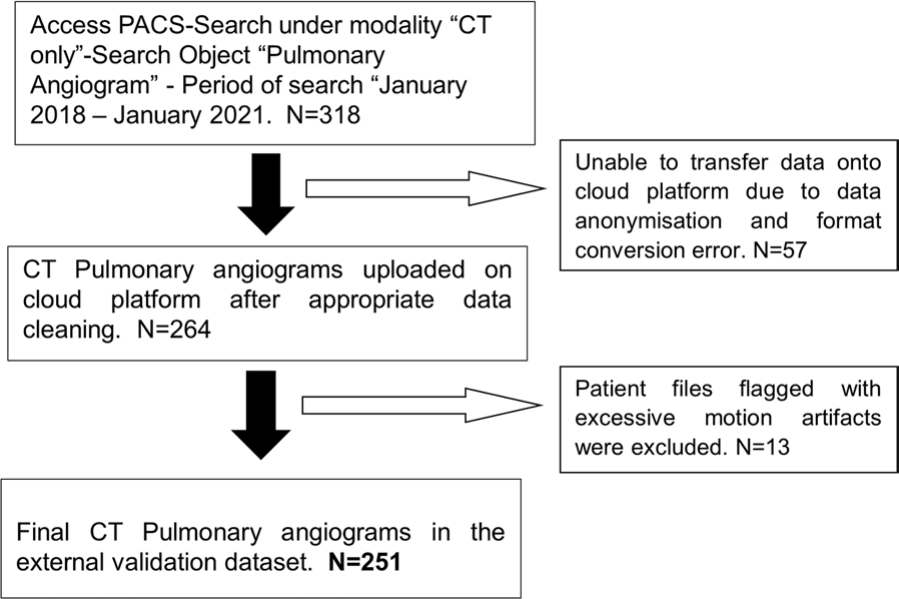
Flowchart illustrating the external validation CT datasets used in the study after implementing inclusion and exclusion criteria

The study was reviewed and approved by the Institutional Review Board of a tertiary care centre. The study was performed in a Health Information Portability and Accountability Act (HIPAA) compliant manner. Due to the retrospective nature of data collection, the need for written informed consent from the individual patients was waived. 251 CTPAs performed at the institution between January 2018 and January 2021 were retrospectively collected. Extraction of datasets was performed utilizing the hospital Picture and Archiving Communication System (PACS). The diagnosis of PE included both clinical information (D-dimer test and echocardiography) and CTPA findings. CTPAs with poor-quality images or motion artifacts were excluded from the study. The flowchart illustrating the selection of scans after the implementation of inclusion and external criteria is represented in Fig. 1. CTPA studies were performed on a 128-slice multidetector Philips Ingenuity CT scanner using the standard protocol. The tube voltage was kept constant at 120 kV, and the scan was performed in the craniocaudal direction with the slice thickness of 0.9 mm for 248 scans, 1 mm for 2 scans, and 2 mm for 1 scan. For contrast injection, the region of interest (ROI) was placed below the carina at the level of the pulmonary trunk with an ROI on the pulmonary artery. For the test bolus, the patient was administered 20 mL of non-ionic contrast with a 10 mL saline chaser at a rate of 5 mL/s. The actual scan procedure was performed with the administration of 60 mL non-ionic contrast with a 100 mL saline chaser at a rate of 5 mL/s.

### Establishing ground truth

A total of 942 CT scans from two open-source datasets, Kaggle FUMPE [27] and RSNA STR Pulmonary Embolism Detection [28], were used for training and internal validation of the model. The external test dataset, consisting of 251 scans, was obtained from a tertiary care centre. To establish the ground truth, three board-certified expert radiologists with 23, 15, and 9 years of experience categorized scans in multiple batches of 25 and highlighted emboli in each slice, if present, using the ITK-Snap software (version 3.8.0). This data was used to calculate the performance of the model at both slice and scan levels.

### Case definition

The radiologists used several indications to identify the presence of PE. For annotating and categorizing the scan as positive or negative, we used the established definitions of PE. Acute PE is defined as (a) the blockage of an artery with the absence of enhancement of the arterial lumen due to a filling defect, which completely obstructs the lumen. Consequently, the artery may enlarge in comparison to neighboring patent vessels. (b) Partial blockage of the artery by a filling defect that partially obstructs the lumen, often producing the “polo mint” sign on sections acquired perpendicular to the long axis of the vessel, and the “railway-track” sign on longitudinal visualization of the vessel. (c) Peripherally located intraluminal defect which forms an acute angle with the arterial wall [29].

### Model details

A 2D segmentation model consisting of U-Net architecture with an Xception encoder was used to detect pulmonary embolism from CTPAs. The input comprised a slice of a CT scan that was preprocessed as mentioned below. The DICOM images were converted to Hounsfield Units, and a window of (40, 400) (Window Level, Window Width) was applied. Subsequently, the images and masks were resized to 512 × 512 voxels for uniformity. The model was trained using Adam Weight Decay as an optimizer and a combination of binary cross-entropy loss (at voxel level) and Dice Loss (at slice level) as loss functions. The output comprised a 512 × 512 image containing the predicted mask. Each voxel of the output image had a value between 0 and 1, indicating confidence in the presence of embolism in that region. Because the model only predicts the masks at the slice level, a method was developed to determine the presence of embolism at the scan level. All voxels having a value greater than 0.5 were assigned the value 1 (corresponding to a ‘positive voxel’), while those with a value less than or equal to 0.5 were assigned the value 0 (corresponding to a ‘negative voxel’). The average number of positive voxels per slice was computed by dividing the total number of positive voxels in a scan by the number of slices in the CT scan. If a CT scan with n slices contains x_1_, x_2_, x_3_… x_n_ positive voxels, then the average number of positive voxels per slice (X) is calculated as:

$$X = \frac{{\sum\nolimits_{i = 1}^{n} {x_{i} } }}{n}$$

**Fig. 2:**
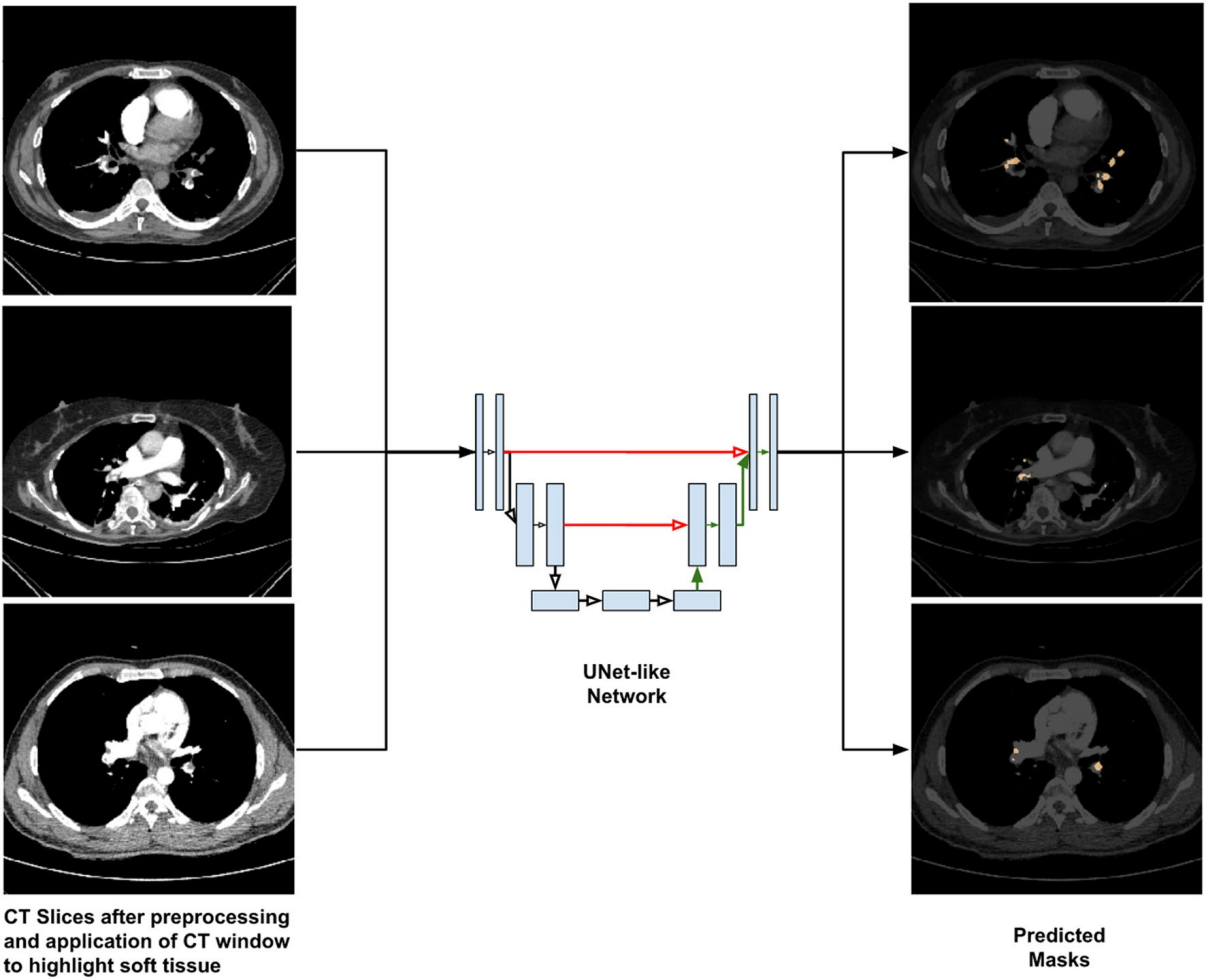
A schematic representation of the PE mask prediction process. Each 2D slice of the CT scan was processed and passed through the model that produced the corresponding mask. The mask highlights emboli. The results of all the slices were combined to form the scan level prediction

The model training was monitored by its performance on a separate validation data set. The model with the lowest validation loss was chosen as the final model. The PE mask prediction process is represented in Fig. 2. The distribution of the number of CT scans and slices in the training, validation, and external test set is given in Table 1.

**Table 1:** Distribution of the number of CT scans and slices in training, validation, and test sets

Set	Training set		Validation set		Test set	
	Number of Slices	Number of Scans	Number of Slices	Number of Scans	Number of Slices	Number of Scans
Negative	167,359	557 (65.4%)	13,217	56 (63%)	140,984	196 (78%)
Positive	14,427	296 (34.6%)	1106	33 (37%)	5183	55 (22%)
Total	181,786	853	14,323	89	146,167	251

### Statistical analysis

The comprehensive evaluation of the model performance on the test set included sensitivity, specificity, PPV, NPV, accuracy, F1 score, and ROC. To measure the variability in these values, we used 95% confidence intervals using the empirical bootstrapping method. To better understand the performance of the model in diagnosing PE, we also calculated confusion metrics on the entire test set.

## Results

### Patient population

**Fig. 3:**
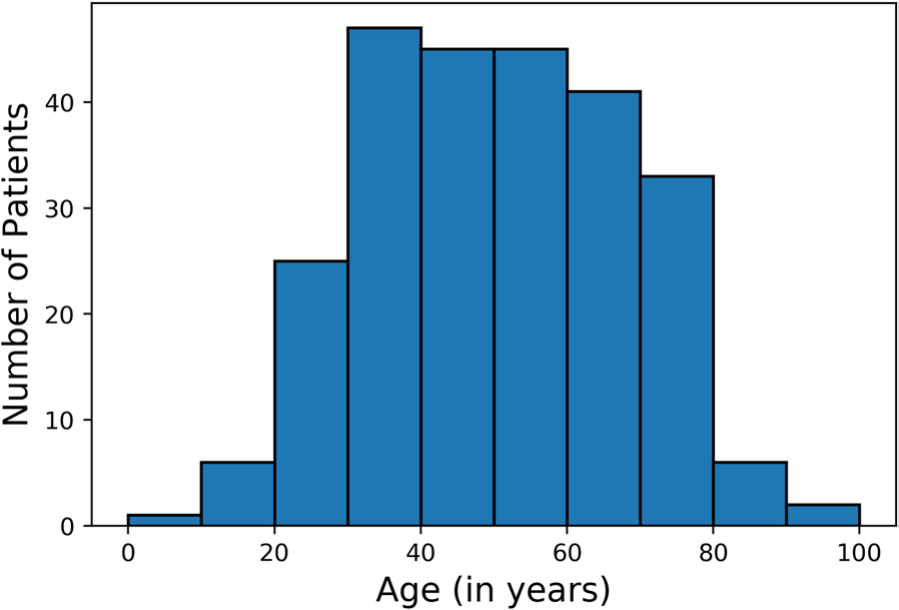
Histogram representing the age distribution of patients included in the study

A total of 251 CTPAs corresponding to 251 patients were used to evaluate the performance of the model on the test set. Among 251 patients, 145 were males and 106 were females, with an average age of 49.7 (S.D. 17.4) years. The age distribution of participants included in the study is represented in Fig. 3. The rate of exams positive for PE was 21.74% (55/251). A total of 3.55% of slices (5183/146167) were positive for PE. There was no missing data.

### Performance of U-Net model

**Fig. 4:**
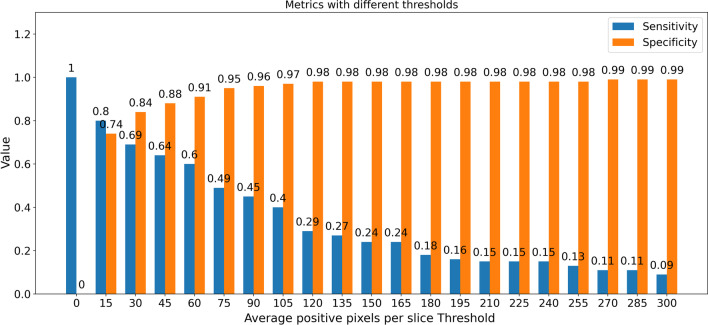
Sensitivity and specificity of the model across different operating points (average positive voxels per slice threshold) on the external test dataset

Table 2 illustrates the segmentation results of the U-Net model on the clinical dataset. The model had a sensitivity of 0.80 [95% CI 0.68, 0.89] and 0.93 [95% CI 0.92, 0.94] at scan level and slice level, respectively. Figure 4 represents the sensitivity and specificity values of the model when the average positive voxels per slice threshold were varied at the scan level. In this study, we set our operating point at a threshold that maximizes both sensitivity and specificity on the external test dataset. Although the threshold > 15 resulted in high specificity values, the corresponding sensitivity values declined. However, in clinical settings, applications are usually tuned to maximize sensitivity in order to minimize false negative rates. Therefore, to calculate the threshold for our model, we calculated the geometric mean of sensitivity and specificity at different thresholds, varying the threshold on steps of 15 from 0 to 300. A threshold of 15 voxels per slice was chosen as the final scan level threshold because it maximized both sensitivity and specificity. This threshold allowed the model to achieve a sensitivity of 0.80 [0.68, 0.89] and specificity of 0.74 [0.68, 0.80].

**Table 2:** Performance metrics of 2D segmentation U-Net model

Metrics	Performance at slice level [95% CI]	Performance at scan level [95% CI]
Sensitivity	0.93 [0.92, 0.94]	0.80 [0.68, 0.89]
Specificity	0.89 [0.88, 0.89]	0.74 [0.68, 0.80]
PPV	0.23 [0.23, 0.24]	0.47 [0.36, 0.57]
Accuracy	0.89 [0.88, 0.89]	0.76 [0.70, 0.81]
F-1 Score	0.37 [0.36, 0.38]	0.59 [0.49, 0.68]
ROC	0.94 [0.936, 0.941]	0.85 [0.78, 0.90]
NPV	0.997 [0.996, 0.997]	0.93 [0.89, 0.97]

**Fig. 5:**
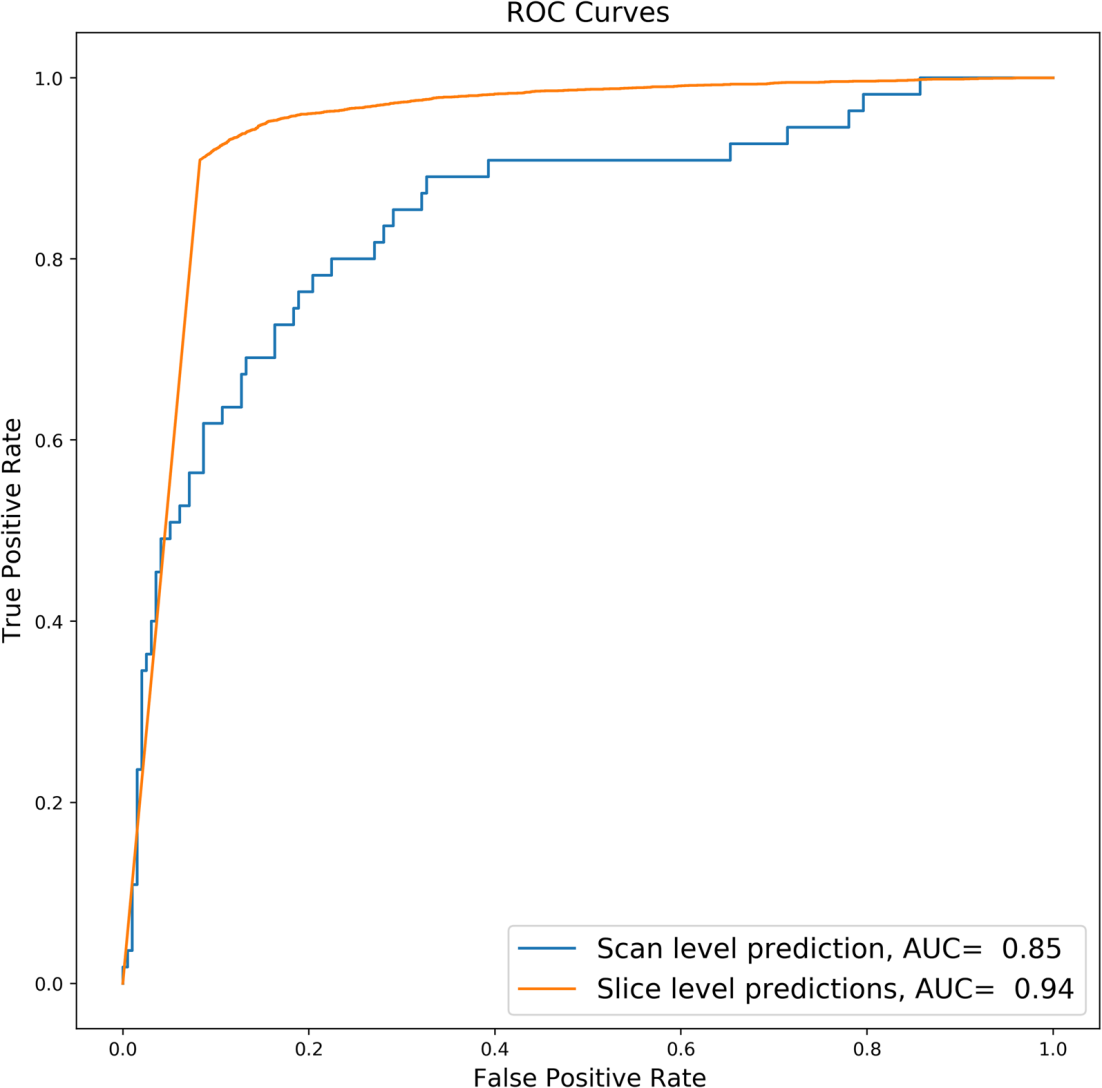
**Performance of 2D segmentation U-Net model.** The AUROC for the external clinical test set

The model achieved an AUROC of 0.85 [0.78, 0.90] and 0.94 [0.936, 0.941] at scan level and slice level, respectively (Fig. 5).

**Fig. 6:**
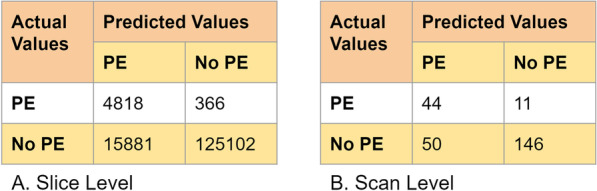
Confusion matrix of PE detection at **a** slice level and **b** scan level

The low number of true positives in the dataset and our choice of operating threshold for high sensitivity resulted in lower PPV (47%) and higher NPV (93%). However, in the clinical setting, improving the sensitivity of the positive cases is more important than PPV (more false positives). The distribution of TPs, FPs, TNs, and FNs for both scan and slice levels are represented in the confusion metrics (Fig. 6).

### Model output

**Fig. 7:**
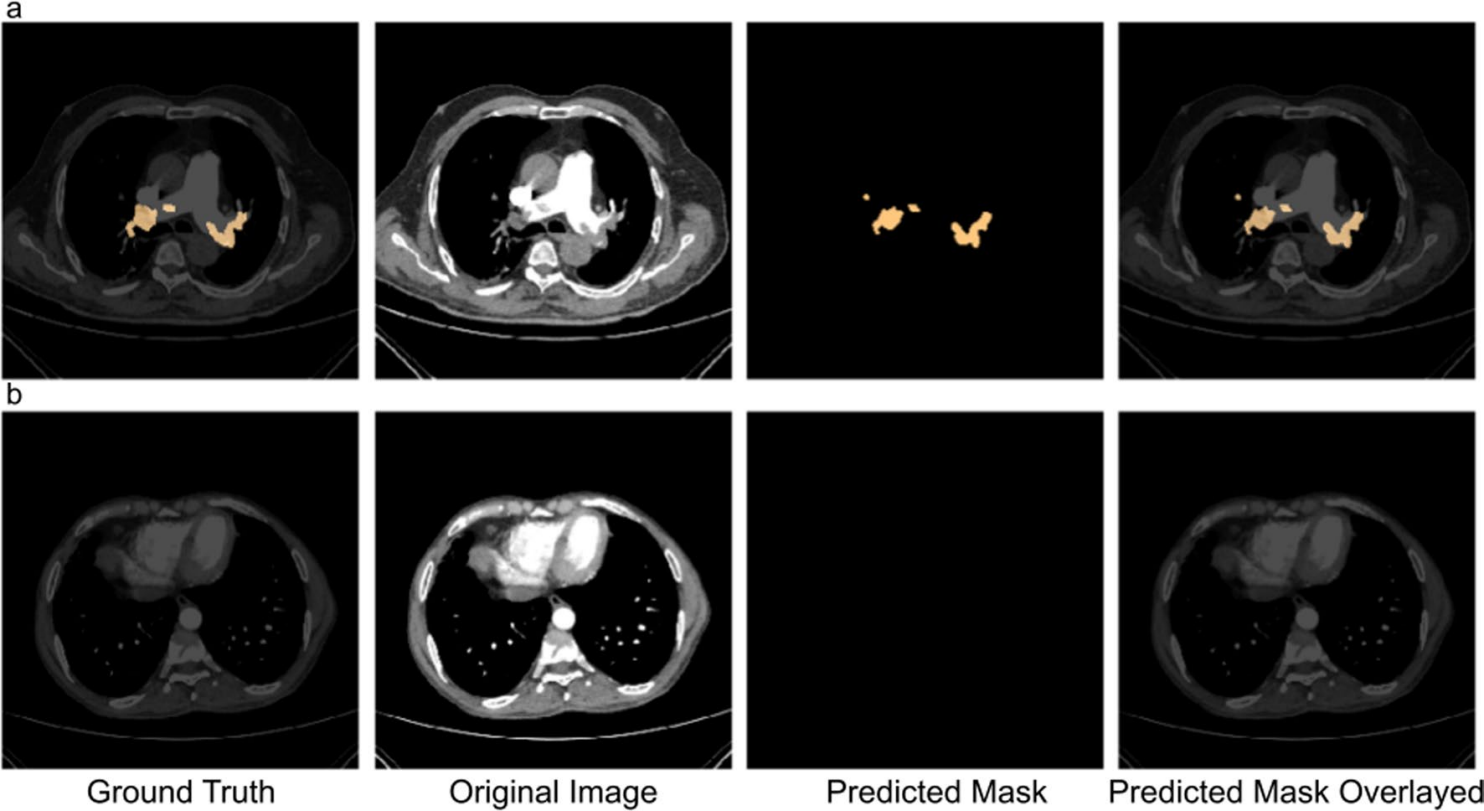
Example of model prediction on a CT slice with **a** embolism present and **b** no embolism

The output of the model contained voxel-wise probability for the input images. The average number of positive voxels per slice was computed by adding all the positive voxels and dividing them by the number of axial slices of the CT scan. An illustration of the same is represented in Fig. 7. The localization of emboli is represented as masks in the CT slice. The model was able to mark the presence of pulmonary embolism in most positive cases. The average segmentation DICE coefficient score per scan for the test set was 0.743 ± 0.155, indicating the high degree of similarity between the reference and the AI-predicted embolus masks.

### Reading time per scan

The standalone model required a mean time of 30.15 s (std. dev: 11.03 s) to process the thin angiography sequences, make inferences, and categorize the scan as positive or negative for emboli.

## Discussion

Suspected pulmonary embolism is a prevalent, and potentially life-threatening condition in emergency patients [30]. Therefore, an accurate and timely diagnosis of PE is critical for improving prognosis. Previous CAD solutions for the automated detection of PE on CTPAs were limited by the low sensitivity of the model [23–26, 31]. Additionally, many of the evaluations were performed on a small dataset with less than 50 cases in the testing set [23, 31–33]. We developed a U-Net-based classification approach for PE detection and tested it on the clinical dataset containing 251 CTPAs. The model achieved high sensitivity, accuracy, and ROC in detecting pulmonary embolism at both slice level and scan level. We also observed that the slice level predictions were slightly better than scan level predictions for AUC, sensitivity, and specificity values. Our model had an AUC of 0.85 at scan level, which is comparable to the previously reported deep learning models. Rajan et al. developed and tested their Convolutional neural network (CNN) model on open-source datasets, reporting an AUC of 0.85 for severe PE and 0.70 for all other PE cases. However, their model was not tested on an independent external dataset [34]. Huhtanen et al. evaluated two deep-learning neural network models for the automated detection of PE from CTPAs using weakly labeled training data. Although one of their models achieved a specificity of 93.5% and sensitivity of 86.6%, they used the same dataset for training and testing [18]. Shi et al. tested a ResNet-based model on a multi-institutional dataset and obtained an AUC of 0.812 [35]. However, the source of their data was not well elucidated. Tajbakhsh et al. evaluated the performance of a CNN model on an internal dataset containing 121 CTPAs with 326 emboli and achieved a sensitivity of 83%. However, their model had a sensitivity of 34.6% on a relatively small test dataset of 20 CTPAs with 133 emboli from the PE challenge [36]. Our model had a slice level sensitivity of 93% and a scan level sensitivity of 80% when tested on an external dataset (not used for model development) with 251 CTPAs (5183 slices containing emboli). Our model matched the performance of the PENet model developed by Huang et al. which achieved an AUC of 0.85 on the external dataset. While they had the advantage of testing their model on two independent external datasets, the maximum sensitivity of their model was 75% [37] at a given threshold. Similarly, Yang et al. reported a sensitivity of 75% for their CNN model, but their test set had only 129 scans (269 emboli) [38].

The use of automated algorithms with a human-in-the-loop approach to triage pulmonary embolism within seconds of a CTPA examination has the potential to be used in clinical practices. It can shorten the time between diagnosis and therapy by highlighting all suspected positive scans for immediate reporting by a radiologist. Li et al. used the computer-aided diagnostic method that effectively improved the detection rate of PE, specifically for intra-arterial embolism above grade 3 [39]. In places where there is insufficient night-time coverage by an experienced radiologist, the algorithm can provide a preliminary diagnosis to the emergency physician, assuring a reliable diagnostic method. Studies have indicated that even in locations with a 24 × 7 radiologist, the quality of reporting is significantly affected, with a discrepancy of nearly 13% between daytime only and night-time faculties in detecting embolism in CTPA studies [40–42]. The use of AI models for the detection of PE can serve as a reliable second opinion for resident radiologists. Additionally, the algorithm can run on a centralized picture archiving and communication system (PACS) or on any edge device in the absence of PACS. The use of edge devices on X-ray, CT, and MRI for diagnostic purposes has already proven to be a huge success, allowing the algorithm to be integrated into the basic system and multiplying its reach [43].

We reported an average DICE coefficient score per scan of 0.743 ± 0.155, which was better than the score reported by Cano-Espinosa et al. on 2D, 2.5D, and 3D networks [44]. Our DL model provides an explainable solution to detect PE, which has the potential to minimize the time required to interpret positive scans and ensure that the peripherally located emboli are not overlooked in cases where a more centrally located embolus is also present.

Our approach testing the 2D segmentation model on the external dataset has certain limitations. Firstly, validation of our model was performed on a single external test dataset. Therefore, future studies will include datasets from multiple clinical institutions to validate the model performance across multiple institutions. Secondly, our model had a low PPV on the external test dataset at both slice and scan levels. This could have resulted because of the low prevalence of the positive CTPAs i.e., 55 scans (though our scans were feature-rich in terms of having a high number of slices containing emboli, i.e., 5183) in our dataset and due to our choice of particularly high sensitivity operating point, which resulted in a lower PPV and higher NPV of 0.47 and 0.93, respectively at scan level. While this approach could falsely label negative scans as positive and increase the urgency of diagnosis, it is important to interpret this in terms of the role this DL model will play in the clinical setting. In a clinical setting, it is acceptable to spend time reassigning a false positive scan into the negative category rather than the disease cost of missing a true positive finding. The model is primarily built for detecting PE and all the scans should be interpreted by a radiologist to provide the final verdict. This human-in-the-loop approach is favored as it reduces the likelihood of missing or delay in reporting a positive scan.

In conclusion, our DL algorithm is on par with the current state-of-the-art DL algorithm and exceeds the performance of some previous DL algorithms. Our approach allows the algorithm to categorize the scan as positive or negative for an embolus in minimal time (mean time of 30.15 s). The mask highlights the region affected by the embolus, thus providing the radiologist with a rapid, reliable, and explainable solution.

## Data Availability

The datasets analyzed in the current study are not publicly available due to ethical restrictions and the proprietary nature of the study but are available from the corresponding author on reasonable request. Please contact richa.pant@deeptek.ai if you want to request the data from this study.

## Abbreviations

AI: Artificial intelligence
AUROC: Area under the receiver operating characteristics
CTPA: Computed tomographic pulmonary angiography
HIPAA: Health information portability and accountability act
PACS: Picture archival and communication system
DICOM: Digital imaging and communications in medicine
CT: Computed Tomography
DL: Deep learning
NPV: Negative predictive value
PPV: Positive predictive value
TP: True positive
FP: False positive
TN: True negative
FN: False negative
CAD: Computer-aided detection
